# Linking Mitochondria to Synapses: New Insights for Stress-Related Neuropsychiatric Disorders

**DOI:** 10.1155/2016/3985063

**Published:** 2016-01-14

**Authors:** Freddy Jeanneteau, Margarita Arango-Lievano

**Affiliations:** Team AVENIR “Stress Hormones and Plasticity”, INSERM U1191, CNRS UMR5203, Institut de Génomique Fonctionnelle, 34094 Montpellier, France

## Abstract

The brain evolved cellular mechanisms for adapting synaptic function to energy supply. This is particularly evident when homeostasis is challenged by stress. Signaling loops between the mitochondria and synapses scale neuronal connectivity with bioenergetics capacity. A biphasic “inverted U shape” response to the stress hormone glucocorticoids is demonstrated in mitochondria and at synapses, modulating neural plasticity and physiological responses. Low dose enhances neurotransmission, synaptic growth, mitochondrial functions, learning, and memory whereas chronic, higher doses produce inhibition of these functions. The range of physiological effects by stress and glucocorticoid depends on the dose, duration, and context at exposure. These criteria are met by neuronal activity and the circadian, stress-sensitive and ultradian, stress-insensitive modes of glucocorticoid secretion. A major hallmark of stress-related neuropsychiatric disorders is the disrupted glucocorticoid rhythms and tissue resistance to signaling with the glucocorticoid receptor (GR). GR resistance could result from the loss of context-dependent glucocorticoid signaling mediated by the downregulation of the activity-dependent neurotrophin BDNF. The coincidence of BDNF and GR signaling changes glucocorticoid signaling output with consequences on mitochondrial respiration efficiency, synaptic plasticity, and adaptive trajectories.

## 1. Introduction

One paramount feature of higher organisms is to learn and adapt to changing environments. It is a matter of survival that requires the brain to convert immediate stimuli into long-lasting changes of neural circuits through alterations of neuronal structure and function [1–3]. Failure to adjust body wide homeostasis in changing environment can cause the organism to follow maladaptive trajectories with presumed pathological consequences [1, 4, 5].

The goal of the present review is to give the cell biologists vision of (mal)adaptive trajectories to stress focusing on the link between the mitochondria and synapses to keep in check “neuronal fitness” in changing environment. If adaptive plasticity necessitates neurons to derail from homeostasis, then disease vulnerability checkpoints could lie in the signaling loops linking mitochondria to synapses. Several studies provide a useful framework for a pathway to neuropsychiatric disorders by the concomitant reorganization of synaptic territories and a dysfunction of mitochondria; but these two responses are often investigated separately [6–10].

Adaptation of metabolism with respect to neural connectivity is presented as a mechanism of neuronal preservation to positive and negative external stimuli. That is, neurons could choose quiescence or growth to fit in their network demands. In particular, the relation between synapses and mitochondria, a pivot of neuronal plasticity, is here discussed, emphasizing on its modulation by the stress hormone, glucocorticoid.

## 2. Linking Mitochondria to Synapse: A Role in Neuronal Plasticity

Several studies indicate that physical proximity between mitochondria and synapses is regulated by neuronal activity [11, 12]. A significant fraction of neuronal mitochondria constantly moves along microtubule networks while the remaining pool is captured where metabolic demand rises [13]. This relation is proportionate, at least in the presynaptic terminals. In the dendrites, the distribution of mitochondria is heterogeneous and difficult to link morphologically with one particular synapse given that mitochondria rarely penetrate into postsynaptic dendritic spines [14]. The functional properties of mitochondria differ in axons and dendrites. For example, mitochondria are more motile in the axons as compared with the dendrites, and there is a greater proportion of highly charged, metabolically active mitochondria in dendrites compared to axons [15]. The following subsections describe mitochondrial functions responding to neurotransmission and likely essential for the plasticity of neuronal networks.

### 2.1. ATP Production

Synaptic activity consumes energy mainly supplied by astrocytes, the biggest reservoir of glycogen in the brain. An elegant study [16] indicated that hippocampal activity induced glycogenolysis, whose end product, lactate, could be used by neurons to produce ATP via glycolytic enzymes that others and we previously found at the synapse [17, 18]. Disruption of lactate transport between astrocytes and neurons impaired the retention of new memories of inhibitory avoidance [16]. Although this study raised the importance of lactate as a signaling molecule or as a source of energy for neurotransmission and behavior, it did not refute the critical role of ATP derived from mitochondria during synaptic plasticity. Neurons rely on mitochondria more than glycolysis to produce ATP [19]. Previous experiments in cultures of hippocampal neurons mostly deprived of astrocytes demonstrated a role for ATP derived from mitochondria in long-term potentiation (LTP) and dendritic spine morphogenesis. For instance, hippocampal neurons, which express dominantly the mitochondrial isoform of the creatine kinase [20], responded to a treatment of exogenous creatine by increasing the number and activity of dendritic mitochondria that coincidently nearly doubled the number of synapses [21].

### 2.2. Calcium Homeostasis

Mitochondria are more than just portable power stations. Mitochondria buffer calcium in the presynaptic terminals and in dendrites after activation of the NMDA receptors by physically coupling with the smooth endoplasmic reticulum (SER) [22, 23]. The capture of cytoplasmic calcium by mitochondria has a modulatory role for neurotransmission at central synapses [24]. The negatively charged electron gradient across the mitochondrial membrane attracts calcium into the matrix via the low-affinity calcium uniport. This phenomenon is reversible so brain mitochondria can store and release calcium [25]. Low levels of matrix calcium levels can control the activity of the oxidative phosphorylation pathway thus changing the rate of ATP production [26, 27]. In contrast, massive accumulation of calcium within mitochondria is cytotoxic and a typical response of glutamate hyperexcitation that can result from disease conditions like stroke or epilepsy [25]. Interestingly, the expression of a cluster of activity-dependent and calcium-sensitive genes can provide neuroprotection through a common process that renders mitochondria more resistant to hyperexcitability [28].

### 2.3. Superoxide Production

Mitochondria produce and release reactive oxygen and nitrogen species that are necessary for synaptic plasticity, learning, and memory [29]. But the abundance of superoxides is extremely damaging for proteins, mitochondrial DNA, and lipids. Therefore, the redox function of mitochondria is also critical for neuronal plasticity during ageing and disease conditions [30]. Remarkably, transgenic mice overexpressing the Superoxide Dismutase 2 (SOD2) exhibit lower levels of mitochondrial superoxides accompanied by normal long-term potentiation (LTP), learning, and memory [31]. This study raises the question whether mitochondrial redox functions are more useful in disease conditions than in health. Indeed, mitochondrial redox functions are breached in disease conditions, causing substantial oxidative stress [32].

### 2.4. Nonapoptotic Caspase Signaling

Mitochondria also activate caspases in dendrites to trigger postsynaptic spine elimination in response to NMDA-dependent long-term depression (LTD) [33]. In particular, the cascade end product caspase-3 is a protease for a broad range of cytoskeletal substrates abundant in dendritic spines (e.g., gelsolin, spectrin, fodrin, and cofilin), in dendritic shafts (e.g., tau), and for glutamatergic/neurotrophic signaling (e.g., calcineurin, AKT) [34]. Consequently, it is hypothesized that mitochondria could participate in LTD by the pruning of synapses. The question remains how synapses to be eliminated are targeted by mitochondrial mechanisms. One possibility is to prevent the spread of caspase signaling to unrelated synapses by the deactivation of the ubiquitin proteasome in hotspots of the dendrites [35]. Alternatively, the expression of caspases is in tight equilibrium with the inhibitors of apoptosis protein (IAP) that suppress generalized caspase activation and neuronal death.

### 2.5. Mitochondrial Maintenance

The size and distribution of mitochondria vary, spanning more than one postsynaptic dendritic spine for elongated mitochondria, while large clusters of neighboring spines can be deprived of dendritic mitochondria. The distribution of dendritic mitochondria depends on the processes of fission/fusion mainly regulated by GTPases (DRP1, OPA1, MFN1, and MFN2) [21, 36]. The fragmentation of mitochondria could serve as a mechanism to cover the needs of distant clusters of spines. In contrast, the elongation of mitochondria could represent a mechanism to increase the ratio of mitochondria per dendrites independently of mitochondrial biogenesis. In terms of plasticity, trains of electrical stimulation elicit slow and prolonged changes in mitochondrial ultrastructure and membrane potential, modifying the proton gradients across mitochondrial membranes necessary to produce ATP [37]. High tetanic stimulation elicits the fragmentation and translocation of mitochondria into enlarged dendritic protrusions in the stimulated region but not in the distant part of the same dendrite [21]. Both rapid and slow mechanisms are likely involved in the maintenance of mitochondrial function upon neuronal activation. Dendritic mitochondria could be rapidly transported where ATP consumption and necessity for energy are particularly high, as it takes place at the active presynaptic terminals [38]. Also, the biogenesis of mitochondria is a slow process, proportionate to neuronal differentiation and growth [39].

## 3. Mitochondria Pay the Price of Synaptic Plasticity

### 3.1. Mitochondria and Synaptic Scaling

Mitochondrial function is closely related to synaptic function. Synaptic scaling is a form of synaptic plasticity that adjusts the strength of all neuronal synapses to the demand of the network [40]. Popular experimental paradigms for inducing synaptic scaling are through the blockade or enhancement of network activity in cultures [41, 42] or sensory deprivation in animal models [43]. For example, the induction of neuronal activity by electrical stimulation or chemical depolarization in cultures decreased the motility and fusion of mitochondria in dendrites via activity-dependent calcium influx [13, 21]. This phenomenon could reflect the synaptic capture of mitochondria as a function of synaptic activity resulting from the pause of mitochondrial transport machinery at hotspots of intracellular calcium signaling. Notably, the calcium-sensing protein MIRO, which links mitochondria to the kinesins microtubule motor proteins, is essential for mitochondrial arrest upon calcium influx through NMDA receptors [44]. In contrast, global suppression of neuronal activity with TTX increased the motility, fusion, and redistribution of mitochondria along dendrites. Therefore, global changes in synaptic activity and glutamate receptor blockers can modulate mitochondrial depolarization and related functions [45]. Likewise, there is a remarkable parallel between the changes in content of synaptic and mitochondrial proteins in the visual cortex of mice reared in the dark compared to mice living under light/dark conditions [46]. Therefore, mechanisms exist for reciprocal regulation of synapses and mitochondria.

Most brain energy is consumed at synapses to maintain ion gradients and support the signaling responses of neurotransmission. The energetic cost of housekeeping neuronal functions is predicted to be higher in the postsynaptic dendrites than in the presynaptic terminals [47]. There are limits to which mitochondria can support trains of neuronal network activation. One example is the repetitive discharges of excitation at hippocampal neurons during epileptic seizures that produce severe mitochondrial dysfunctions, eventually resulting in neuronal death [48]. Modeling experiments indicate that synaptic depotentiation is likely desirable to support neuronal survival when energetic stores are limited [19]. Therefore, negative feedback mechanisms may have evolved to suppress synaptic potentiation that would drain ATP stores upon high frequency stimulation. One putative target that can sense low metabolic state is the AMP-dependent protein kinase (AMPK), whose activation impedes the transition from the early synaptic potentiation phase to the long-term synaptic potentiation phase [49]. So neurons evolved protective cellular mechanisms for adapting synaptic function with metabolism. By pushing neuronal plasticity beyond the limits of homeostasis, one could learn in the pathways that link mitochondria to synapses how to promote adaptation. The next section will focus on the synaptic and metabolic adaptation to traumatic stress.

### 3.2. Mitochondria and the Allostatic Load Model

Neuronal networks are capable of adaptive responses because they can reach a novel state of physiological stability each time homeostasis is challenged. This phenomenon termed allostasis requires the setting of new metabolic, housekeeping, and plasticity parameters in accordance with the external demands. It is the nature of the allostatic load that should define the amplitude of adaptation to reach without killing the cells [50]. Therefore, signaling loops between mitochondria and synapses are anticipated to be essential for coping with the allostatic load [51]. The allostatic load model has been extensively studied in the context of stress because of its comorbidity with numerous human neuropsychopathologies and, experimentally, it is admitted that stress is a starting point for the development of chronic disease trajectories. Studies of traumatic stress early in life emphasized the important roles of the age and timing at exposure, the nature, intensity, and duration of the stressor for developing enduring metabolic dysfunction, and neuropsychopathologies in adulthood [52]. Consequently, there is not one but multiple trajectories of adaptation to stress set by the interaction of genetics, lifestyle, and the environment coming in different flavors. Remarkably, twin studies revealed that distinct adaptive trajectories could also evolve from organisms with the same genetic background and history [53–55]. These studies naturally raised the question as to why some individuals are vulnerable to disorders linked to stress (e.g., major depressive disorders, anxiety, and posttraumatic stress disorders) whereas others are not despite similar stressful experiences [56].

Stress, when chronic and uncontrollable, is a major risk factor of neuropsychopathologies. Underlying mechanisms are complex and cannot be ascribed to a single genetic or environmental factor, but they usually result in dysfunctional metabolism and neuronal connectivity of cortical and subcortical micro- and macrocircuits. So, neuropsychiatric disorders induced by stress most likely arise from complex interplay of genetics and environment. The level of stress hormones reflects perceived changes of the external world (e.g., threat, reward, and novelty) by readily diffusing throughout the circulation, virtually accessing all cells of the organism within the same time scale. For this reason, the physiology and adaptive capacity of the hormonal stress response mediated by the hypothalamo-pituitary-adrenal (HPA) axis has been intensively studied [57]. It is admitted that physiological feedforward and feedback neuroendocrine loops control the HPA axis as a function of the frequency and dose of internal and external stimuli [58]. Alterations of these activation and deactivation loops have been reported in patients suffering from stress-related neuropsychiatric disorders and in animal models [59, 60]. One important question remains if the allostatic overload of stress relies on mechanisms of plasticity that link mitochondrial function to synaptic plasticity. From the characterization of such mechanisms could emerge novel modulators of stress-related neuropathologies.

## 4. Glucocorticoids Are Fast and Slow Acting Modifiers of Synapses and Mitochondria

Stress and glucocorticoids have potent but complex effects on neurotransmission, learning, and memory. The typical inverted “U” shape response to glucocorticoids and stress depends on the dose, duration, and context at exposure. The convergence of several mechanisms is likely to explain such biphasic responses. First, it could reflect the circadian and ultradian oscillations of glucocorticoids secreted in the bloodstream [57]. Second, it could reflect the interaction of the stress-elicited norepinephrine and glucocorticoids signaling that produces a response distinct from individual pathways during the early learning and memory phases, as a function of stressor intensity and duration [61–63]. Third, it could reflect the dynamics of dendritic spines that can be modified by the changes of glucocorticoids levels in the bloodstream, like the circadian- or stress-mediated changes in oscillatory secretions. Glucocorticoid-induced spine formation is rapid and temporally dissociated from spine survival and elimination, which are slow and required the synthesis of new gene products [64]. Correlation studies suggest that dendritic spine patterning and mnemonic effects on procedural learning afforded by glucocorticoids depend on the dose, duration, and context at exposure [65].

Biphasic modulatory effects of glucocorticoids have been described on mitochondrial function as well [66]. For example, stimulation of primary neurons with corticosterone increased, in time- and dose-dependent manner, mitochondria calcium holding capacity, membrane potential, and redox function. These effects depended also on the context at exposure, producing neurotoxicity or neuroprotection, respectively, with or without a cotreatment of kainic acid [66]. These findings recapitulate that of glucocorticoids on synaptic plasticity, indicating that common or converging pathways control synaptic and mitochondrial functions. There is evidence that such relation exists in vivo as well. Serial section electron microcopy of synapses in the amygdala of animals experiencing fear conditioning revealed that fear learning increased the size of the postsynaptic density, the number of presynaptic docking vesicles, and the number of mitochondria compared to naïve controls and the conditioned inhibition group that learned safety conditioning [67]. This result is in agreement with the glucocorticoid-dependent hypertrophic effects of amygdala neurons in the fear-conditioning paradigm [58]. Other studies indicated that mitochondria ultrastructure and bioenergetics capacity are altered in neurons or brain regions, which demonstrated synaptic pathology (e.g., bipolar disorder, major depressive disorders, schizophrenia, and Alzheimer's disease) [68–71]. In vitro studies indicated that glucocorticoids increased mitochondrial calcium holding capacity, mitochondrial membrane potential, and mitochondrial oxidation rapidly and durably depending on the dose [66]. Glucocorticoid receptors (GR) are required for modulating mitochondria as well as dendritic spine turnover. The former involved the translocation of cytoplasmic GR-BCL2 complexes into the mitochondria [66]; the latter required membrane bound GR presumably localized at the synapse in the motor cortex [64] and previously described in the postsynaptic dendrites of amygdala neurons [72].

**Figure 1:**
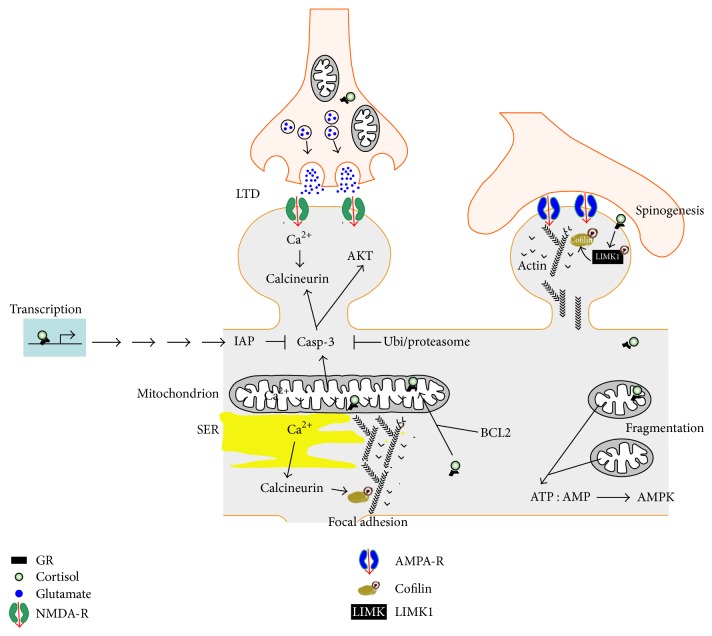
The mitochondria-synapse signaling loop is modulated by glucocorticoids. Acute and moderate glucocorticoid peaks rapidly promote the formation of new dendritic spines via a membrane GR coupled to the activation of the LIMK1-cofilin pathway. In contrast, glucocorticoid-mediated spine elimination is delayed and requires the transcription of new gene products. GR is present at pre- and postsynaptic membranes, in the cytoplasm, the nucleus, and the mitochondria. The exact mechanisms and series of molecular events are unknown. Trains of electrical stimulation impose an intense energy demand that can result in mitochondrial fragmentation if unmet, thereby increasing the ATP : AMP ratio, the activation of the AMP-sensing kinase to signal the local decrease of energy stores. New mitochondria can be captured in a calcium-dependent manner where energy stores are low. The levels of intracellular calcium determine whether or not to activate the calcium-dependent phosphatase calcineurin, which can be disruptive for the focal adhesion of mitochondria by dephosphorylating cofilin, impacting on the polymerization of the acting cytoskeleton tethering membranes to the mitochondria. Additionally, synaptic pruning can result from NMDAR-dependent LTD, calcium-dependent cytochrome c release whose end product is the activated caspase-3. Caspase-3 exerts local nonapoptotic effects via a broad spectrum of synaptic substrates. To this end, caspase-3 activity is retained at hotspots thanks to transcription of inhibitors of apoptosis proteins (IAP), some of which are GR-regulated genes, and by a constitutive active ubiquitin-proteasome degradation system from which caspase-3 can only be protected within the hotspots. Select transcriptional targets of GR have been involved in the regulation of respiration, mitochondrial uncoupling, and elongation, the dynamics of the actin cytoskeleton and synaptic plasticity.

The exact roles of GR at the synapse or in mitochondria are uncertain (Figure 1). However, new evidence points toward a role for GR signaling derived from mitochondria and synapses on the dynamics of the actin polymerization between G-actin and F-actin cytoskeleton via the LIMK1-cofilin pathway [64, 73]. The assembly and disassembly of G-actin to F-actin are critical for many cellular processes, including cell motility, migration, dendritic spine morphogenesis, endocytosis, and focal adhesion of mitochondria at pre- and postsynaptic membranes [74]. Interestingly, the broad-spectrum serine/threonine phosphatase calcineurin, essential for functional and structural neuronal plasticity, can be activated on both ends of the mitochondria-synapse pathway. Calcium entry via the NMDA receptors activates calcineurin, and depolarization of mitochondrial membranes results in calcineurin activation, perhaps through the proteolytically activated caspase-3 pathway [75]. Other possible pathways involve the transcriptional regulation of the nuclear and mitochondrial genes bearing GR responsive elements (GRE), demonstrated to participate in the process of oxidative phosphorylation (OXPHOS) in mitochondria [76].

## 5. Effects of Chronic Stress on Mitochondria and Synapses

Aberrant mitochondrial function and metabolite levels (e.g., ATP) have been documented in patients suffering from stress-related disorders [77, 78], suggesting that people harboring “low power” mitochondria could be more vulnerable to stress. The link between depression and rare polymorphisms in genes encoding for mitochondrial proteins (*TFAM, BCL2, TOMM40, and mitochondrial DNA*) provided valuable mechanistic insights although the sample size is small [77]. The impact of these mutations coincides with the reported increased fragmentation of neuronal mitochondria, the decrease of mitochondrial membrane potential, the increase of ROS production, and the decrease of mitochondrial respiration and calcium buffering capacity after exposition to chronic stress or chronic administration of synthetic glucocorticoids [6]. One consensus is that prolonged glucocorticoid signaling could damage mitochondrial functions whereas acute effects could facilitate mitochondrial functions. Speculatively, the accumulation of damaged mitochondria could erode the bioenergetics capacity of neurons particularly at risk during repetitive and intense challenges. Consistent findings were reported in the brain and peripheral cells of patients with major depressive disorders [79]. Interestingly, the brain regions featuring metabolic deregulation and dysfunctional mitochondria are those also exhibiting changes in neuronal connectivity and neurotransmission at least in animal models [80, 81].

Protective mechanisms driven by the transcription of nuclear genes evolved to limit damage of dangerous challenges. One such putative mechanism of resilience to stress is mitochondrial uncoupling operated by the family of proteins UCP (UCP1–5) [82]. The expression of UCPs, in particular UCP2, increases upon chronic stress resulting in a collapse of mitochondrial membrane polarization, dissipating the proton gradient to produce heat rather than ATP [83]. One advantage is to reduce the production of superoxides, to decrease locally the ATP : AMP ratio, resulting in the activation of the AMPK negative feedback pathway to reduce synaptic potentiation and increase metabolism where needed [84–86]. Another consequence of mitochondrial uncoupling is the elongation of mitochondria, which increases calcium-buffering capacity and counteracts mitophagy thereby enhancing mitochondrial functions not related to OXPHOS. The shape and size of the mitochondria are highly variable depending on the processes of fission and fusion. For example, Mfn1, Mfn2, SOD1, and SOD2 are downregulated by chronic exposure to corticosterone and stress [87]. Remarkably, the knockout of UCP2 exacerbated the depressive-like phenotypes in a mouse model of chronic inflammation featuring disrupted glucocorticoid levels [88]. Therefore, promoting mitochondrial uncoupling and restoring metabolic functions of mitochondria are potential disease modifying strategies to cope with chronic stress.

## 6. Effects of Antidepressants on Mitochondria and Synapses

Successful antidepressant therapies eventually increase mitochondrial functions and the number of functional synapses where brain activity is reduced like in the prefrontal cortex [89]. The best-known examples are calorie restriction and voluntary exercise that enhance mitochondrial function with demonstrated antidepressant effects. These studies indicated that mitochondrial dysfunction and cortical atrophy could be reversed by lifestyle modifications [87, 90, 91]. The adaptation capacity of mitochondria and synapses to voluntary exercise was almost null in UCP2 knockouts, suggesting that a mitochondrial mechanism related to UCP2 function is required for appropriate bioenergetics adaptation of neurons to the increased neuronal plasticity induced by voluntary exercise [91].

The effect of tricyclic antidepressants was also evaluated on metabolism and mitochondrial function. In vivo, the incorporation of radiolabelled deoxyglucose is reduced in several brain regions after a single injection of clomipramine in an animal model [92, 93] and in the human brain upon acute treatment with lithium [94]. Specifically, acute treatment of cells in vitro with serotonin reuptake inhibitors (SSRI), noradrenaline serotonin reuptake inhibitors (SNRI), and monoamine oxidases inhibitors (MAOI) deteriorates mitochondrial bioenergetics capacity [77]. Ketamine also impaired mitochondrial bioenergetics capacity in vitro and in vivo [95, 96]. Only one study [97] reported the amelioration of OXPHOS with paroxetine. Although most antidepressants reduced metabolic functions of mitochondria, the redox functions increased in conditions that facilitate synaptic functions [77, 98]. Ketamine, for example, ameliorated the ratio of NADH/NAD+ by increasing SOD activity in the rat brain [96] and in stem cell derived human neurons in culture [99]. Speculatively, deterioration of OXPHOS functions by antidepressants could underlie the many side effects of treatment, whereas beneficial effects could involve the amelioration of mitochondrial redox functions [100].

Strategies to boost mitochondrial bioenergetics and/or redox functions alone or in combination with existing therapies improved symptoms of stress-related disorders in human and animal models. For example, 3 of 4 clinical trials with creatine monohydrate in combination with antidepressant drugs accelerated the efficacy of treatment modalities without improving the maximal therapeutic benefits in an overall small sample size [101–104]. Remarkably, the use of antioxidants (e.g., Zinc, N-acetyl cysteine, vitamin E, and coenzyme Q10) as supplements in the treatment of neuropsychiatric disorders also provided some promising results in major depression and bipolar disorder [105]. Another potentially interesting target of the mitochondria-synapse loop is AMPK because it is activated by intensive voluntary exercise and AMPK triggers the uncoupling mitochondrial response [106]. Remarkably, the antidepressant effects of exercise and ketamine depended on the activation of AMPK at least in the hippocampus [107, 108]. Additionally, activation of AMPK with the small molecule agonist AICAR was sufficient and more efficient than exercise for promoting antidepressant-like effects in diabetic mice [109]. Finally, the brain-derived neurotrophic factor (BDNF) is a necessary target of successful antidepressant therapies, whose signaling modulates mitochondria biogenesis, respiration and redox functions [39, 100, 110, 111], AMPK signaling, synaptogenesis, and neurotransmission [112] as a function of glucocorticoid levels frequently deregulated in patients suffering from stress-related neuropsychiatric disorders [113–118]. This important point raises the possibility that glucocorticoid and BDNF signaling pathways are dependent and both necessary for the efficacy of antidepressant therapies [119, 120].

## 7. Context-Dependent Glucocorticoid Signaling: BDNF Is Context!

Glucocorticoids effects on mitochondria and synapses depend on the dose, duration, and context at exposure [121]. The effects of dose and duration recapitulated by the acute and chronic stress paradigms were discussed earlier. The context at exposure such as learning at the time of glucocorticoid oscillation peak could produce unique effects, temporally restricted, that are the results of the coupling of neuronal activity with glucocorticoid signaling in select neuronal networks [64, 121]. For example, a single injection of glucocorticoids within minutes of motor skill training enhanced motor learning. In contrast, similar dose administered hours before or after has no effects on behavior, on dendritic spine patterning, or on neurotransmission [64, 122, 123].

Contextual glucocorticoid signaling is determined by (1)* the temporal window*: survival of learning-associated new spines and behavioral performance required that circadian peaks and troughs remain normal during the week after learning to consolidate the acquired memory in the form of clusters of dendritic spines; and by (2)* the spatial window*: plasticity occurred in the amygdala when learning fear conditioning, in the hippocampus CA1 when electrically stimulating the Schaffer collaterals, and in the motor cortex when learning motor skill abilities. If true, glucocorticoid-mediated spine plasticity during motor skill learning should be specific of the motor cortex. Out of the context of motor learning, glucocorticoids enhance the turnover of dendritic spines in all the cortical regions tested (sensory S1, S2, frontal cortex, and motor M1, M2), but these new spines are short-lived and cannot survive at distant time point [65]. Only glucocorticoid signaling within the context of motor learning elicited the survival of clusters of dendritic spines for long periods of time, perhaps to store new information [64]. Such coincidence detection of glucocorticoids signaling with neuronal activity in the cortical circuits involved in learning could be essential for the encoding of the memory trace. It means that glucocorticoid therapies based on chronic nonpulsatile treatment could cause spine patterning defects and cognitive disabilities by altering the coincidence [125]. Importantly, the recent discovery that memory does not correlate with the long-term survival of dendritic spines in the hippocampus suggests that cortical regions operate differently [126]. In this case, the role of hippocampal neurogenesis, which is absent from the cortex, is obviously an important aspect for future studies.

**Figure 2:**
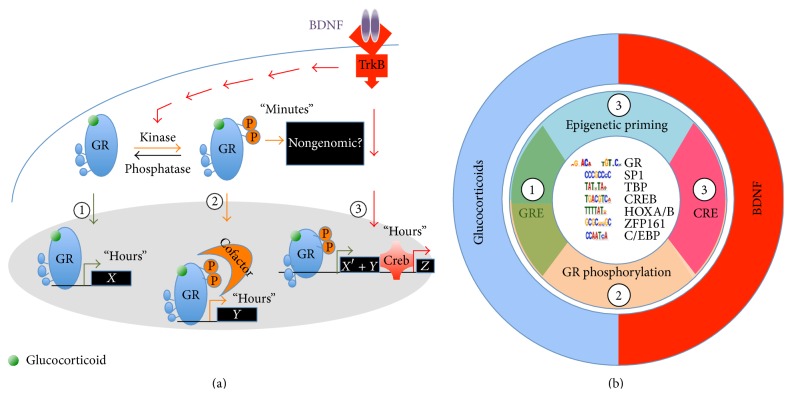
Neurotrophic priming of glucocorticoid signaling. (a) The coincidence of BDNF and glucocorticoid signaling triggers rapid and slow effects different from the sum of genes regulated by individual pathways.* Pathway-1*: glucocorticoids impact the expression of GR-regulated genes.* Pathway-2*: BDNF-induced GR phosphorylation could foster the recruitment of cofactors that change transcriptional output.* Pathway-3*: BDNF/TrkB-responding genes plus epigenetic priming at locus previously unexposed to the activated GR. (b) In the center are listed the most represented genomic DNA ligands bound to GR upon stimulation of cortical neurons with BDNF and dexamethasone. The interplay of BDNF and glucocorticoid signaling uses the mechanisms of epigenetic priming as well as GR phosphorylation to specify the range of targets. Image adapted from [124].

What could be the molecular mechanism of coincidence? The convergence of two signaling pathways is anticipated: (1) one known, the glucocorticoid, and (2) one unknown but activity-dependent. If GR signaling is central for glucocorticoid spine plasticity and mitochondrial function, BDNF signaling is a suitable candidate, pivot of activity-dependent synaptic plasticity and mitochondrial function [112, 114, 127, 128]. Other putative candidate modulators of GR signaling have also been described (e.g., norepinephrine, inflammatory cytokines, FGF, and IGF1) [123, 129–132]. BDNF signaling via its receptor TrkB can rewrite GR-mediated gene expression signature [133]. This effect resulted from a composite response of (1) additive effects perhaps due to epigenetic priming and of (2) unique effects at a select cluster of genes that only responded to the coincidence of BDNF and GR signaling (Figure 2).

Mechanistically, BDNF signaling elicits the phosphorylation of GR at serines 134 and 267, which fosters cofactor recruitment like CREB1 to promote a novel gene expression signature [133]. Among target genes,* CRH* expression is decreased in the hypothalamus and increased in the amygdala by GR signaling [58, 134]. In the context of BDNF signaling in the hypothalamus, GR signaling used the CREB coactivator CRTC2 as coincidence detector to activate or suppress CRH expression with physiological consequences on the neuroendocrine responses of stress in mice [135]. In the context of contextual fear learning, glucocorticoid-induced memory consolidation of inhibitory avoidance required coincident BDNF signaling in the hippocampus [136]. The effects of concomitant BDNF and glucocorticoids signaling depended on the dose, duration, and context at exposure [120].

## 8. Glucocorticoid Resistance in Stress-Related Neuropsychiatric Disorders

Glucocorticoid resistance is a state of reduced tissue responsiveness to glucocorticoids [137, 138] observed in disorders of chronic inflammation, during aging, in neuropsychiatric disorders, and in neurodegenerative diseases. Major hallmarks of these conditions are the elevated levels of circulating cortisol (due to a lack of GR-mediated negative feedback control) and reduced BDNF levels [116, 119, 139, 140]. The question remains whether deficits of BDNF can impair context-dependent GR signaling that may set the stage for developing GR resistance. Transcriptomic analyses of GR-regulated genes in depressed patients compared to healthy controls indicated that the expression of select clusters of genes (e.g., anti-inflammatory) is impaired while others are not affected [141]. Such selectivity at target genes indicates that mechanisms of glucocorticoid resistance are complex, resembling a loss of context-dependent GR signaling.

Several mechanisms of GR resistance have been proposed: (i) a decreased expression of GR that has been reported, as much as the increased expression of inactive splice variants [142], (ii) rare mutations in the GR that can cause a generalized glucocorticoid resistance syndrome [143], (iii) impaired epigenetic control of GR-regulated gene expression by HDAC2 [144, 145], and (iv) the transport of GR into mitochondrial matrix which fails in cells resistant to the biological effects of glucocorticoids [146, 147]. Remarkably, glucocorticoids modulate mitochondrial biogenesis and OXPHOS pathway by regulating the transcription of the mitochondrial genome [148, 149]. Amidst the complexity, context-dependent GR signaling in the form of BDNF-priming of GR signaling could unify the neurotrophic hypothesis [150] and the glucocorticoid hypothesis [151] of depression by modulating mitochondrial responses.

## 9. Conclusions

Glucocorticoid resistance is a common feature of ageing, neurodegenerative diseases, and neuropsychiatric disorders, conditions that can be ameliorated with BDNF mimetic therapies (e.g., electroconvulsive shock, deep brain stimulation, antidepressant drugs, and exercise).  Table 1 summarizes the effects of BDNF, glucocorticoids, stress, major depressive disorders, and antidepressant therapies on mitochondrial and synaptic functions. In theory, chronic antidepressant treatment, BDNF signaling, and context-dependent GR signaling should be associated with improved mitochondrial functions and positive neuroplasticity. In reality, antidepressants improve the redox function while deteriorating the bioenergetics capacity, which could explain the mixed benefits of such treatments. On the contrary, inherited and environmental factors that diminished BDNF and GR functions can aggravate the progression of neuropsychiatric disabilities. This is the case of highly penetrant gene mutations in* BDNF* (Val66met, [152, 153]),* TrkB* (Y722C, [154]),* FKBP51* (rs1360780 [155]),* 5-HTTLPR* (rs6354 and rs2020936 [156, 157]), and* COMT* (Val158Met [158]). Likewise, chronic stress, disrupted circadian rhythms, chronic neuroinflammation, and chronic high dose glucocorticoid therapies are associated with “low power” mitochondria and negative neuroplasticity.

BDNF and glucocorticoids are essential for cognitive functions and stress coping. Deregulation of their activities is a risk factor for developing psychiatric disorders by impairing synaptic plasticity of brain circuits mediating reward learning, while bolstering circuits mediating aversion learning. Future strategies aiming at boosting mitochondrial functions will identify new targets against GR resistance, perhaps at intersection of BDNF and glucocorticoid signaling pathways in specific circuits.

## Figures and Tables

**Table 1:** Summary of the cellular and physiological effects of BDNF, glucocorticoids, stress, major depressive disorders, and antidepressant therapies.

	BDNF	Glucocorticoids	Stress	Major depression	Antidepressant therapies
Synaptic structure	Formation, maintenance	Formation, elimination, and maintenance^#¶^	Formation, elimination^#¶^	Elimination^¶^	Formation, maintenance^#¶^
Synaptic function	Potentiation	Potentiation, depression^#¶^	Potentiation, depression^#¶^	Depression^¶^	Potentiation^#¶^
Mitochondria structure	Biogenesis^*∗*^	Augmented/diminished^#*∗*^	Augmented/diminished^#^	Diminished^¶^	Augmented/diminished^#*∗*^
Mitochondria energetics function	Augmented ^¶^	Augmented/diminished^#*∗*^	Augmented/diminished^#*∗*^	Diminished^¶^	Augmented/diminished^#*∗*^
Mitochondria redox function	Augmented^¶^	Augmented/diminished^#*∗*^	Augmented/diminished^#^	Diminished^¶^	Augmented^#*∗*^
Learning and memory	Augmented^¶^	Augmented/diminished^#^	Augmented/diminished^#^	Diminished	Augmented^#^
Despair and anxiety	Diminished^¶^	Augmented/diminished^#^	Augmented/diminished^#^	Augmented	Diminished^#^

#: effect depends on the dose, duration, and context at exposure.

¶: effect is specific of the brain region.

*∗*: effect demonstrated in cultured cells.
